# Potassium sorbate induces developmental and microbiome changes in *Drosophila melanogaster* with attenuated trans-generational toxicity

**DOI:** 10.3389/fmicb.2026.1783630

**Published:** 2026-03-23

**Authors:** Yuling Dong, Beibei Du, Changjian Xie, Shujing Zhang, Qiuxiang Pang, Desheng Zhang

**Affiliations:** 1 School of Life Sciences and Medicine, Institute of Anti-aging and Regenerative Medicine Research, Shandong University of Technology, Zibo, Shandong, China; 2 State Key Laboratory of Vascular Homeostasis and Remodeling, NHC Key Laboratory of Cardiovascular Molecular Biology and Regulatory Peptides, Beijing Key Laboratory of Cardiovascular Receptors Research, Health Science Center, School of Basic Medical Sciences, The Institute of Cardiovascular Sciences and Institute of Systems Biomedicine, Peking University, Beijing, China; 3 School of Materials Science and Engineering, Shandong University of Technology, Zibo, Shandong, China

**Keywords:** *Drosophila melanogaster*, growth and development, gut microbiota, potassium sorbate, trans-generational effect

## Abstract

Potassium sorbate (PS) is a widely used antimicrobial additive employed as a preservative in food, cosmetics, and animal feed. Early childhood represents a critical developmental window characterized by rapid growth, immune system maturation, gut microbiota establishment, and physiological homeostasis development, which may be particularly vulnerable to chemical exposures (during which chemical exposures may exert heightened impacts). Nevertheless, the effects of PS on childhood development and gut microbiota remain poorly understood, and its potential trans-generational effects have yet to be elucidated. In this study, we employed *Drosophila melanogaster*, an established model for studying evolutionarily conserved aspects of development, metabolism, and host–microbiota interactions, to systematically evaluate PS toxicity across multiple parameters: developmental dynamics, gut microbiota composition, gene regulation in adulthood, and trans-generational effects. Our findings demonstrate a dose-dependent biphasic response: while low-dose PS exposure (25 mg/L) accelerated larval pupation and adult emergence, suggesting a potential growth-promoting effect, high-dose exposure (≥ 500 mg/L) significantly delayed development and reduced adult lifespan (observed in females at 1,000 and males at 500–1,000 mg/L). Notably, trans-generational analysis revealed persistent developmental delays in the F1 generation, with offspring of 1,000 mg/L-exposed parents showing prolonged larval pupation time despite normal adult emergence timelines, indicative of partial developmental recovery. Mechanistically, high-dose PS disrupted endocrine signaling and altered expression of key developmental pathway regulators (*EcR*, *InR*, *TOR*, and *E74B*). These transcriptional changes were largely reversible in offspring, further supporting a similar gradually wash out recovery. While gut microbiota remained stable in exposed parents, offspring of 1,000 mg/L-exposed flies had significant microbiome alterations, highlighting clear trans-generational dysbiosis. This study provides new evidence that PS exposure during a sensitive developmental period in *D. melanogaster* can perturb development and gut microbial homeostasis across generations, offering insights relevant to understanding how early-life chemical exposures might influence conserved biological processes in higher organisms.

## Graphical Abstract

**Figure:**
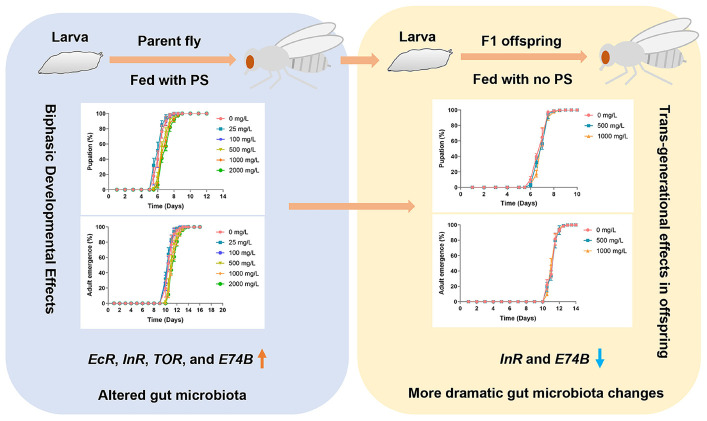
The effect of potassium sorbate (PS) on development of early life stage of *D. melanogaster*, gut microbiota, trans-generational effects and the potential mechanisms. PS exerts biphasic and transgenerational effects in *D. melanogaster*. Low-dose PS (25 mg/L) accelerates larval development, while high-dose exposure (≥ 500 mg/L) delays pupation, reduces lifespan, and disrupts endocrine signaling (*EcR*, *InR*, *TOR*, *E74B*). Although parental gut microbiota remains stable, F1 offspring exhibit developmental delays and significant microbial dysbiosis—revealing trans-generational toxicity of a common food preservative.

## Introduction

Microbial growth-induced food spoilage remains a major global challenge, making shelf-life extension a critical priority for the food industry (Machado et al., 2020; Dehghan et al., 2018; Snyder et al., 2024). As essential additives, food preservatives enhance product preservation through multiple antimicrobial mechanisms, including disruption of microbial cell membrane integrity, inhibition of key enzymatic activities, and interference with cellular metabolic processes (Yardimci et al., 2022; Qian et al., 2024). However, the potential health risks associated with synthetic preservatives have raised persistent consumer concerns, posing a major constraint on industry development.

Potassium sorbate (PS, C_6_H_7_KO_2_), the potassium salt of sorbic acid, is a broad-spectrum synthetic food preservative used globally. This odorless compound typically appears as a white crystalline powder or scaly crystals and is significantly more soluble in water than sorbic acid, and its solubility is temperature-dependent. As an effective antimicrobial agent, PS exhibits inhibitory activity against a wide range of microorganisms, making it particularly valuable for food preservation applications. Its preservative efficacy extends to diverse food categories including vegetables, fruits, meat, seafood,

candies, baked goods, beverages, fermented sauces (e.g., soy sauce), and pickled products (Silva and Lidon, 2016; Xiao et al., 2024; Dehghan et al., 2018). PS is generally recognized as safe by the United States Food and Drug Administration (FDA) (FDA, 2017). According to Annex II of Regulation (EC) No 1333/2008, sorbic acid and its salts (sorbates) are authorized as food additives in the European Union, with maximum permitted levels ranging from 20 to 6,000 mg/kg in solid foods and from 20 to 6,000 mg/L in beverages and other liquid foods (EFSA Panel on Food Additives and Nutrient Sources added to Food [ANS], 2015). The Joint FAO/WHO Expert Committee on Food Additives (JECFA) has established an acceptable daily intake (ADI) for sorbates, expressed as sorbic acid, of 0–25 mg/kg of body weight (Carocho et al., 2014). For a 60 kg adult, the upper end of this range corresponds to a daily intake of 1.5 g of sorbic acid equivalents, representing the level considered safe for lifelong consumption. Studies have reported that actual human exposure to PS ranges from 17 to 48% of the ADI (Reddy et al., 2015; Zouaoui et al., 2024).

Despite typical usage levels generally being well below ADI thresholds, the rising global consumption of processed foods driving by market expansion and lifestyle changes raises concerns. Accumulative exposure to PS through multiple food categories (e.g., baked goods, dairy, beverages) may approach safety limits in high-consumption situations (Amirpour et al., 2015; Yazdanfar et al., 2023). While PS has been extensively evaluated for acute toxicity, its chronic toxicity and potential developmental effects, particularly those associated with early-life exposure and long-term consequences, remain insufficiently characterized.

Early childhood is a stage of rapid growth and development, representing a critical developmental window for establishing immune homeostasis, a stable and healthy gut microbiota, and physiological homeostasis. Children are more vulnerable to chemical exposure, even doses lower than those deemed safe for adults may harm them, leading to long-term adverse effects on their physical and psychological development (Scott and Pocock, 2021; Dourson et al., 2002). However, little is known about the effect on children growth and development, and the gut microbiota development. Therefore, it is crucial to evaluate the safety of PS during early-life stage, and whether the toxicity of PS could pass to the next generation.

The gut microbiota represents a complex ecosystem of microorganisms inhabiting the gastrointestinal tract, comprising bacteria, archaea, fungi, viruses, and protozoa (Clemente et al., 2012). The latest research estimates that the ratio of gut microorganisms to human body cells is approximately 1:1 comprising over 1,000 bacterial species (Sender et al., 2016). The gut microbiota contributes to host health through diverse physiological roles, including nutrient absorption and harvesting energy (Turnbaugh et al., 2006), protecting against pathogens (Pickard et al., 2017), regulating host immunity (Rooks and Garrett, 2016), and strengthening gut integrity or shaping the intestinal epithelium (Fassarella et al., 2021). Gut microbiota dysbiosis has been associated with more than 30 human diseases, including inflammatory bowel disease (Matsuoka and Kanai, 2015), obesity (Zhao, 2013), diabetes (Gurung et al., 2020), non-alcoholic fatty liver disease (Aron-Wisnewsky et al., 2020), neuro-degenerative disorders (e.g., Alzheimer’s disease and Parkinson’s disease) (Mincic et al., 2024), neurodevelopmental conditions (e.g., autism spectrum disorder) (Warner, 2019), and various cancers (Vivarelli et al., 2019; Sekirov et al., 2010). The growing recognition of the relationship between gut microbiota and host health has prompted urgent reassessment of food preservatives, particularly their effects on gut microbial ecology. Notably, while EFSA’s related guidance emphasized the need for microbiome safety data, current regulatory frameworks lack specific guidelines for evaluating preservative effects on gut microbiota (Merten et al., 2020; Moreno et al., 2024). The potential disruption of gut microbial composition and function by PS remains poorly characterized.

Therefore, in this study, we used *Drosophila melanogaster* to model the impact of early-life PS exposure on developmental processes relevant to growth and gut microbiota establishment. PS was administered throughout the larval stage, the critical window for nutrient absorption and microbial colonization, and its effects were assessed in newly eclosed adult flies (< 8 h post-eclosion), a time point chosen to capture the initial composition and functional profile of the adult gut microbiota prior to dietary perturbations. We also evaluated potential trans-generational effects by examining developmental and microbial outcomes in the F1 offspring of PS-exposed parental flies.

*D. melanogaster* is widely used in toxicological research due to their short life cycle, genetic tractability, and ethical advantages. Importantly, key aspects of its biology, including intestinal structure, gut microbial composition at the phylum level, and core signaling pathways such as insulin/TOR and ecdysone/EcR, are evolutionarily conserved with mammals. The larval stage, characterized by rapid growth and metabolic programing, thus provides a tractable system to gain initial insights into developmental toxicity mechanisms relevant to human health. By evaluating PS-induced physiological, microbial, and generational changes across developmental stages, this study aims to address critical gaps in food additive safety assessments.

## Materials and methods

### *D. melanogaster* husbandry

For this study, we utilized the wild-type *D. melanogaster* Canton-S-iso3A strain, sourced from the Bloomington *Drosophila* Stock Center at Indiana University (Stock #9516, Bloomington, IN, United States). The flies were reared on a standard yeast-sucrose-cornmeal diet, with each liter of medium containing 25 g of yeast, 40 g of sucrose, 42.4 g of maltose, 66.825 g of cornmeal, 9.18 g of soybean meal, 6 g of agar, 0.5 g of sodium benzoate, 0.25 g of nipagin, and 6.875 mL of propionic acid. The experimental treatment diets were prepared by supplementing the standard diet with PS at concentrations of 0, 25, 100, 500, 1,000, or 2,000 mg/L. These concentrations were selected according to the global regulatory agencies’ maximum permitted levels and the acceptable daily intake (ADI) established by JECFA (25 mg/kg bw/day), and the maximum permitted levels of sorbic acid – sorbates in foods According to Annex II of Regulation (EC) No 1333/2008 (20–6,000 mg/kg or mg/L), as well as the toxicity study in other researches (Xiao et al., 2024; Peng et al., 2019). Our dose selection links real-world exposure levels with toxicological test standards. The PS used in this study was obtained from Aladdin (Shanghai, China) with a purity of 99%.

To evaluate trans-generational effects, mated female flies chronically exposed to 500 or 1,000 mg/L PS were transferred to egg-laying chambers containing fresh control diet (PS-free) for a 24-h oviposition period. Embryos were then reared to adulthood on standard PS-free medium to assess F1 developmental outcomes. Fresh food was prepared weekly and stored at 4°C to prevent it from drying out. The flies were maintained under controlled conditions at 25°C and 65% humidity, with a 12-h light-dark cycle.

### Developmental time measurement

The duration of pupariation (larval-to-pupal transition) and eclosion (pupal-to-adult emergence) was systematically quantified in PS-exposed flies through daily monitoring. Pupariation events were identified by two morphological markers: (1) anterior spiracle eversion and (2) cuticle tanning, while adult eclosion was confirmed by complete emergence from the pupal stage.

### Adult body weight quantification

The body weight of 1-day-old adult flies was measured using groups of 10 sex-matched individuals per replicate to ensure sufficient biomass for detection. For each PS treatment group, flies were anesthetized briefly with CO_2_, transferred to a pre-weighed 1.5 mL microcentrifuge tube, and weighed collectively using an analytical balance (Mettler Toledo MS105DU, ± 0.0001 mg accuracy, Zurich, Switzerland). After subtracting the tube weight, the average weight per fly was calculated. Six independent biological replicates (each comprising 10 flies) were analyzed per condition.

### Survival analysis

For survival analysis, three PS treatment groups were established by supplementing the standard cornmeal-yeast-agar diet with 0 mg/L (control), 500 mg/L, or 1,000 mg/L PS. Newly eclosed adult flies (< 8 h post-eclosion) were briefly anesthetized with CO_2_ for no more than 30 s to facilitate randomly assigning to single-sex groups (males or females). Flies were then allowed to recover for at least 24 h under standard rearing conditions before assignment to different groups and initiation of survival assays. For each treatment and sex combination, 10 independent vials (24 mm diameter × 95 mm height) were established as the experimental units, each containing 20 newly eclosed flies. All vials were maintained under controlled environmental conditions at 25 °C, 65% relative humidity, and a 12:12 h light–dark cycle. Survival was monitored by counting dead flies every 2–3 days at the individual level, and flipping flies into fresh vials every 5 days to maintain optimal conditions. Kaplan–Meier survival curves were constructed using individual fly lifespan data, and differences between groups were assessed by the Mantel–Cox log-rank test (Obata et al., 2018).

### *16S rRNA* gene amplicon analysis

#### Sample collection and DNA extraction

Adult flies (< 8 h post-eclosion) from both the control (0 mg/L PS) and treatment (1,000 mg/L PS) groups were collected and sequentially surface-sterilized through rinsing in 50% (v/v) household bleach for 30 S and 70% ethanol for 1 min, respectively. They were then extensively washed with phosphate-buffered saline (PBS) before dissection (Obata et al., 2018). Each treatment group has four biological replicates, for each biological replicate, guts from 30 to 35 flies were dissected in sterile PBS using sterile forceps. The trachea, malpighian tubules, and crop were carefully removed, and the remaining gut tissue was collected in PBS on ice. The gut samples were subsequently stored at -80°C in 1.5 mL microcentrifuge tubes for further analysis.

Genomic DNA was extracted using a BioBase animal tissue DNA extraction kit (M2012-01, Chengdu, China) following the manufacturer steps. The gut tissues were first thoroughly homogenized for at least 1 min using a tissue grinder (Tiangen, OSE-Y20, Beijing, China) with a pestle (Tiangen, OSE-Y001, Beijing, China). The homogenate was then transferred to a 2 mL tube containing 600 μL of Lysis/Wash Buffer (BioBase, M2012-01, Chengdu, China). This mixture was subsequently transferred to a new 2 mL tube containing 30 μL of BioBase Tissue Beads (BioBase, M2012-01, Chengdu, China). Following lysis and centrifugation steps, including elution with Wash Buffer and Spin Column Wash Buffer, the beads were air-dried at room temperature. DNA was finally eluted using Elution Buffer (BioBase, M2012-01, Chengdu, China). DNA concentrations were measured using a Nanodrop 2000c (Thermo Scientific, Waltham, MA, United States). The final DNA concentrations ranged from 208.6 to 321.3 ng/μL, with A260/A280 ratios between 1.81 and 1.97 and A260/A230 ratios between 2.03 and 2.19. DNA size and integrity were assessed by electrophoresis on a 1% agarose gel. Based on the measured concentrations, DNA samples were diluted to 1 ng/μL using sterile water for subsequent analyses.

#### Illumina NovaSeq sequencing

Sequencing was provided by Wekemo Tech Group Co., Ltd. (Shenzhen, China). The genomic *16S rRNA* genes from the distinct V3-V4 regions were amplified using specific primers 341F (5’-CCTAYGGGRBGCASCAG-3’) and 806R (5’-GGACTACNNGGGTATCTAAT-3’), which included unique barcodes. Each PCR reaction was conducted in a total volume of 30 μL, containing 15 μL of Phusion^®^ High-Fidelity PCR Master Mix (New England Biolabs, United States), 1 μL of forward primer (1 μM/μL), 1 μL of reverse primer (1 μM/μL), 10 ng of template DNA (10 μL), and 3 μL nuclease-free H_2_O. No-template PCR controls (nuclease-free water substituted for DNA in amplification reactions) were run for every batch of extractions and PCRs to assess contamination and bias. The thermal cycling conditions were as follows: an initial denaturation at 98°C for 1 min, followed by 30 cycles of denaturation at 98°C for 10 s, annealing at 50°C for 30 s, and elongation at 72°C for 30 s. A final extension was carried out at 72°C for 5 min.

PCR products were mixed with 6 × DNA loading dye and separated by electrophoresis on a 2% agarose gel prepared in 1 × TAE buffer, with electrophoresis carried out in 1 × TAE running buffer. The PCR products were pooled in equimolar ratios, and the resulting mixture was purified using the Universal DNA Purification Kit (TianGen, China).

Sequencing libraries were prepared using the NEBNext^®^ Ultra™ DNA Library Prep Kit (New England Biolabs, United States) following the manufacturer’s protocol, purified V3–V4 amplicons from individual samples were pooled prior to library construction, and adapters with unique dual indexes were ligated directly to the pooled amplicons without any post-ligation PCR enrichment. The quality and size distribution of the constructed libraries were assessed using an Agilent 5400 TapeStation System (Agilent Technologies Co., Ltd., United States), confirming the expected fragment size (∼450–500 bp, including adapters). Libraries that met the quality criteria were then sequenced on the Illumina NovaSeq platform, producing 250 bp paired-end reads.

#### Bioinformatics analysis

The bioinformatics analysis was conducted following the “Atacama Soil Microbiome Tutorial” from Qiime2docs ^1^ with customized scripts. Briefly, raw data FASTQ files were imported into the format which could be operated by QIIME2 system using qiime tools import program. Demultiplexed reads were processed in QIIME2 using the DADA2 plugin. Primers (338F/806R) were removed with q2-cutadapt. Forward and reverse reads were both truncated at 249 bp, filtered for max expected errors ≤ 2.0, denoised, merged, and subjected to *de novo* chimera removal to generate an amplicon sequence variant (ASV) table (Callahan et al., 2016). Taxonomic classification of ASVs was performed using the QIIME2 feature-classifier plugin, aligning sequences against a pre-trained GreenGenes 13_8 99% OTU reference database, trimmed to the V3**–**V4 region targeted by the 338F/806R primer pair (Bokulich et al., 2018). Mitochondrial and chloroplast sequences were filtered out using the QIIME2 feature-table plugin.

Appropriate methods including ANCOM, ANOVA, Kruskal Wallis, LEfSe, and DEseq2 were employed to identify the bacteria with different abundance among samples and groups (Love et al., 2014; Mandal et al., 2015; Segata et al., 2011). Diversity metrics were calculated using the core-diversity plugin in QIIME2. Alpha diversity indices, such as Chao1 richness estimator, Faith’s PD, Shannon diversity index, and Simpson index were computed to estimate microbial diversity within individual samples. Beta diversity analyses, including Bray-Curtis dissimilarity, unweighted UniFrac, and weighted UniFrac distances, were used to examine structural variations across microbial communities and visualized via principal coordinate analysis (PCoA) and non-metric multidimensional scaling (NMDS) (Vázquez-Baeza et al., 2013). Supervised Partial Least Squares Discriminant Analysis (PLS-DA) was applied using the plsda function in the R package mixOmics to reveal microbiota variations among groups (Rohart et al., 2017).

The potential KEGG Ortholog functional profiles of the gut microbial communities were predicted using Phylogenetic Investigation of Communities by Reconstruction of Unobserved States (PICRUSt) (Langille et al., 2013). Linear Discriminant Analysis (LDA) Effect Size (LEfSe) analysis was performed using the LEfSe module available on the online Galaxy platform.

#### Related genes quantitative reverse transcription-PCR

Newly eclosed adult flies (< 8 h post-eclosion) reared on either control (0 mg/L PS) or treatment (1,000 mg/L PS) diets were selected for transcriptomic analysis. For each biological replicate, 15–20 whole flies were homogenized in TLB Buffer (BioBase, N1002-01) using a tissue grinder (Tiangen, OSE-Y20) equipped with a pestle (Tiangen, OSE-Y001). Total RNA was extracted following the manufacturer’s instructions for the RNA extraction kit (BioBase, N1002). RNA concentrations were quantified using a NanoDrop 2000c spectrophotometer (Thermo Scientific, Waltham, MA, United States), and 1 μg of total RNA from each sample was reverse-transcribed into cDNA using a RevertAid First Strand cDNA Synthesis Kit (Thermo Fisher Scientific, #K1622, United States).

Quantitative real-time PCR (qRT-PCR) was performed on a Roche LightCycler 480 II real-time PCR cycler (Roche, Basel, Switzerland) using 2 × Q3 QuantiNova SYBR Green II PCR Master Mix (22204-1, TOLOBIO). Each reaction was conducted in triplicate to ensure reliability of the results. The relative mRNA expression levels were normalized to the housekeeping gene *rp49*, and fold changes were calculated relative to the control group. Primer sequences for qRT-PCR are detailed in Supplementary Table 1.

#### Statistics

Development time, survival curves, and Mantel-Cox log-rank test were analyzed using GraphPad Prism 7 software (GraphPad Software, La Jolla, CA, United States). Comparisons between two groups were performed using two-tailed Student’s *t*-tests. For multiple comparisons, one-way analysis of variance (ANOVA) followed by Tukey’s *post-hoc* test was applied to identify significant differences among groups. All graphs depict the mean ± 1 standard deviation (SD), unless stated otherwise. Statistical significance is indicated by asterisks: * for *p* < 0.05 and ** for *p* < 0.01.

## Results

### Developmental time measurement

**FIGURE 1:**
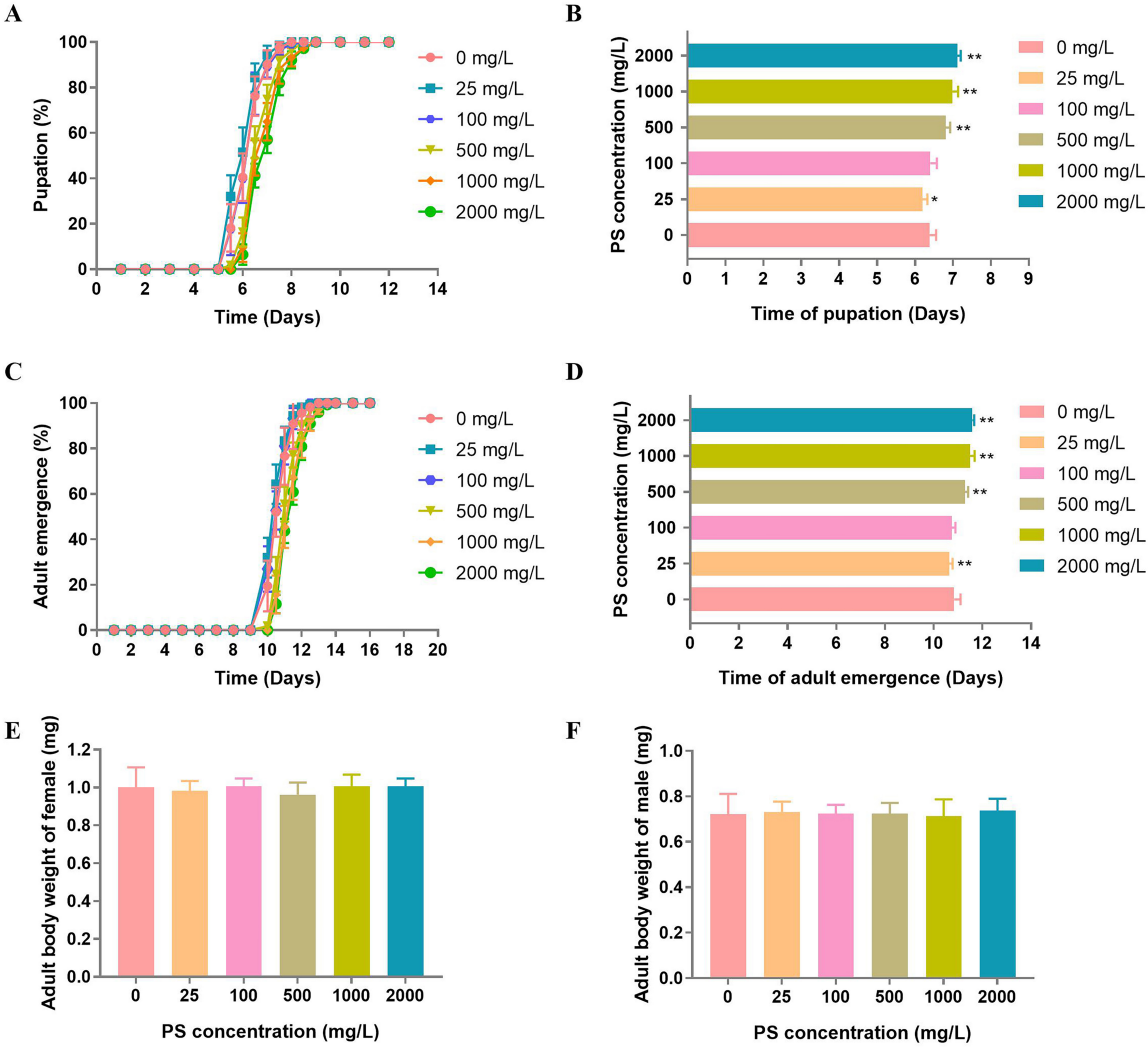
The development time of flies exposed to PS. **(A)** Pupation time of flies exposed to PS at concentrations of 0, 25, 100, 500, 1,000, and 2,000 mg/L. **(B)** Mean time of the pupation for flies exposed to PS at concentrations of 0, 25, 100, 500, 1,000 and 2000 mg/L. **(C)** Adults emergence time of flies exposed to PS at concentrations of 0, 25, 100, 500, 1,000 and 2,000 mg/L. **(D)** Mean time for flies exposed to PS at concentrations of 0, 25, 100, 500, 1,000 and 2,000 mg/L. **(E)** Adult body weight of female flies exposed to PS at concentrations of 0, 25, 100, 500, 1,000 and 2,000 mg/L. **(F)** Adult body weight of male flies exposed to PS at concentrations of 0, 25, 100, 500, 1,000, and 2000 mg/L. *p < 0.05, **p < 0.01.

Regarding pupation timing, a biphasic dose-response relationship was observed following PS exposure (Figures 1A,B). Flies exposed to 25 mg/L PS exhibited significantly accelerated pupation [reduced by 5.75 ± 1.94 h, *p* = 0.009, Hedges’ *g* = –1.26, 95% CI (–2.10, –0.40)] compared to controls (0 mg/L PS). In contrast, higher PS concentrations (≥ 500 mg/L) significantly delayed pupation (prolonged by 10.28 ± 2.69 h at 500 mg/L, *p* < 0.0001, Hedges’ *g* = 2.96, 95% CI [1.82, 4.07]; 14.46 ± 3.62 h at 1,000 mg/L, *p* < 0.001, Hedges’ *g* = 3.82, 95% CI [2.57, 5.04]) relative to controls.

Consistent with the pupation timing results, adult emergence exhibited a comparable biphasic response to PS exposure (Figures 1C,D). Flies treated with 25 mg/L PS showed significantly accelerated adult emergence (reduced by 5.66 ± 1.68 h, *p* = 0.04, Hedges’ *g* = –0.93, 95% CI [–1.74, –0.10]) relative to controls (0 mg/L PS). Conversely, exposure to higher PS concentrations (≥ 500 mg/L) resulted in dose-dependent emergence delays (500 mg/L: 10.67 ± 3.17 h, *p* < 0.0001, Hedges’ *g* = 2.02, 95% CI [1.02, 3.00]; 1,000 mg/L: 15.79 ± 4.44 h, *p* < 0.0001, Hedges’ *g* = 2.88, 95% CI [1.73, 4.01]).

Notably, no significant differences in adult body weight were observed between control group (0 mg/L PS) and any of the other PS-exposed group, even at the highest concentration tested (2,000 mg/L PS), in either males or females (Figures 1E,F). This finding demonstrates that while PS exposure dramatically alters developmental timing, it does not significantly affect somatic growth in adult *D. melanogaster*, even at extreme concentrations.

**FIGURE 2:**
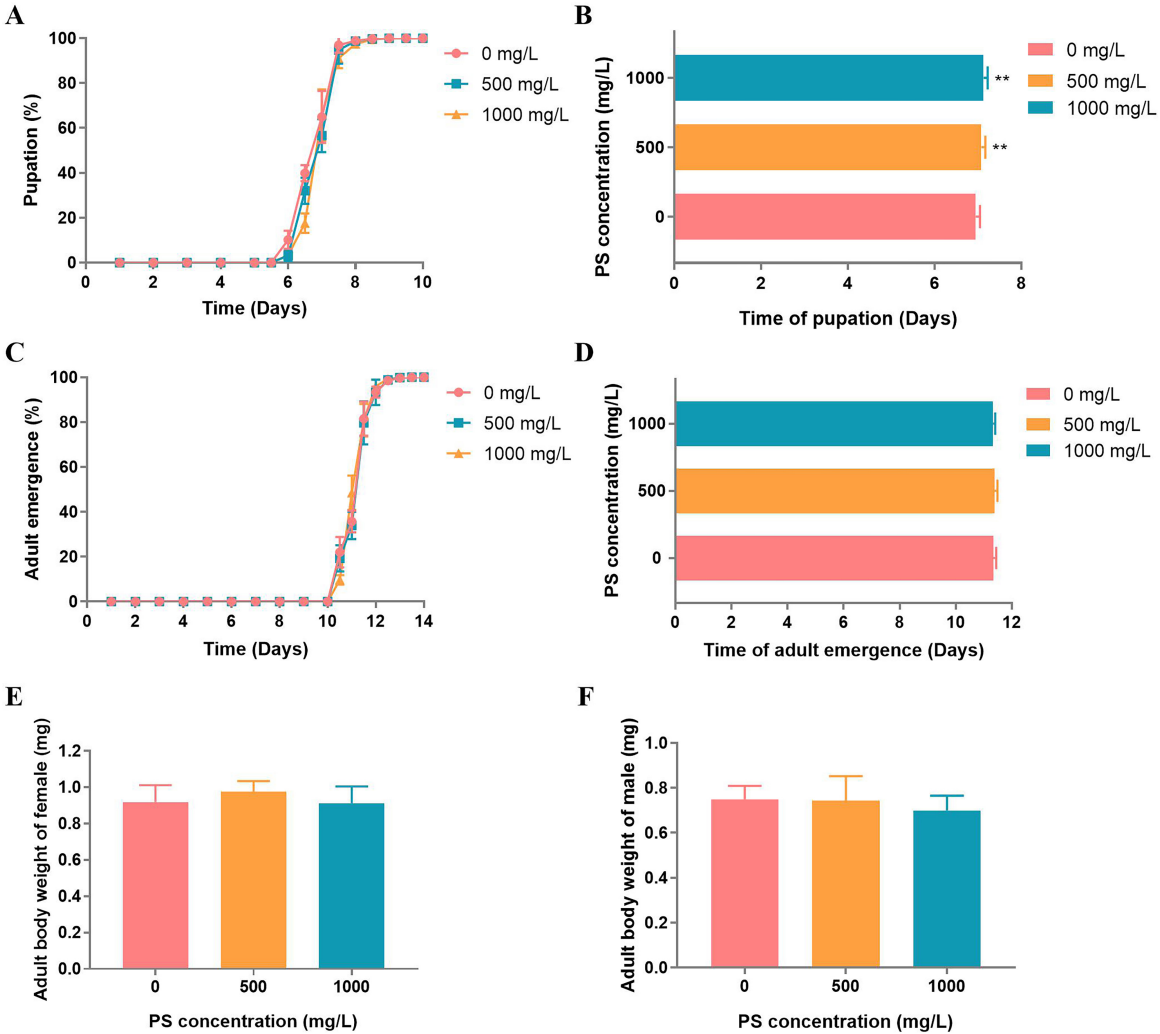
Effects of PS on *D. melanogaster* offspring larval development. **(A)** Pupation time of offspring larvae exposed to PS at concentrations of 0 mg/L, 500, and 1,000 mg/L. **(B)** Mean time of offspring larvae exposed to PS at concentrations of 0, 500, and 1,000 mg/L. (C) Adult emergence time of offspring larvae exposed to PS at concentrations of 0, 500, and 1,000 mg/L. **(D)** Mean time of the adult emergence time of offspring larvae exposed to PS at concentrations of 0, 500, and 1,000 mg/L. **(E)** Adult body weight of female offspring exposed to PS at concentrations of 0 mg/L, 500, and 1,000 mg/L. **(F)** Adult body weight of male offspring exposed to PS at concentrations of 0, 500, and 1000 mg/L. **p < 0.01.

Trans-generational analysis demonstrated concentration-dependent effects of parental PS exposure on offspring development (Figure 2). Compared to controls (0 mg/L PS), offspring from PS-exposed parents showed significantly prolonged pupation times at both 500 mg/L [*p* = 0.008, Hedges’ *g* = 1.25, 95% CI (0.34, 2.14)] and 1,000 mg/L PS [*p* = 0.0005, Hedges’ *g* = 1.87, 95% CI (0.84, 2.88)] (Figures 2A,B). Notably, these developmental delays were not accompanied by changes in adult emergence timing, which remained unaffected across all concentrations (500 mg/L: *p* = 0.54, Hedges’ *g* = 0.27, 95% CI [–0.61, 1.15]; 1,000 mg/L: *p* = 0.68, Hedges’ *g* = –0.22, 95% CI [–1.09, 0.65]; Figures 2C,D). Similarly, no significant differences in adult body weight were observed between offspring of 1,000 mg/L PS-exposed flies and controls (Figures 2E,F). These results indicate that parental PS exposure selectively affects developmental timing in offspring without influencing somatic growth or metamorphosis completion.

### Survival analysis

**FIGURE 3:**
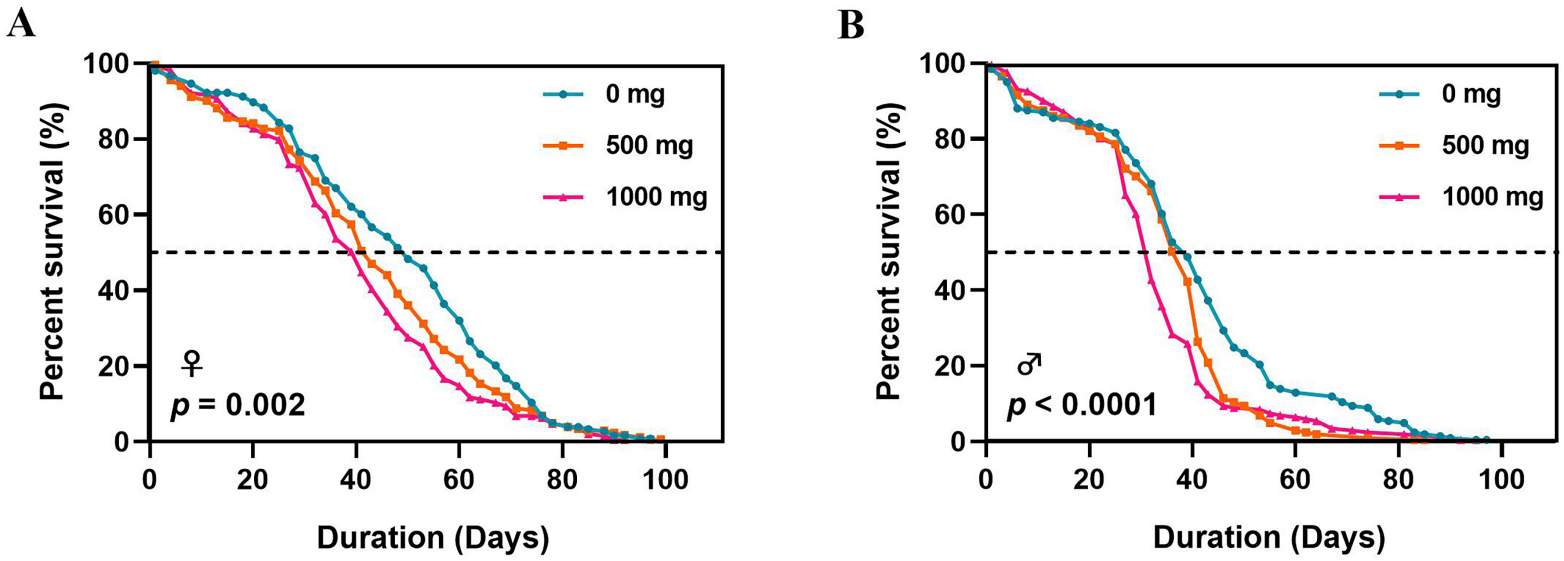
Survival curves of PS-exposed *D. melanogaster*. (A) Lifespan of female flies exposed to PS at concentrations of 0 mg/L, 500 mg/L and 1,000 mg/L. (B) Lifespan of male flies exposed to PS at concentrations of 0 mg/L, 500 mg/L and 1,000 mg/L.

PS exposure induced significant, dose-dependent reductions in female *D. melanogaster* lifespan (Figure 3A and Table 1). Compared to flies in control group (0 mg/L PS; median lifespan = 50 days), flies’ median lifespan decreased by 14% (to 43 days; *p* = 0.0003, HR = 1.20, 95% CI [0.99, 1.47]) at 500 mg/L PS and by 18% (to 41 days; *p* < 0.0001, HR = 1.40, 95% CI [1.46, 1.71]) at 1000 mg/L PS. Notably, the flies’ lifespan difference between 500 mg/L and 1,000 mg/L exposures was also significant (*p* = 0.0357, HR = 1.96, 95% CI [0.95, 1.41]), demonstrating progressive toxicity with increasing PS concentration. These findings suggest that PS exposure causes cumulative physiological damage that scales with dosage.

**TABLE 1:** Statistics for survival curves.

Group	Group	Total n. of Flies	Median (% Change)	Log-Rank
Female				
0	500	200	50	*p* = 0.0003
	1,000			*p* < 0.0001
500	1,000	200	43 –14%)	*p* = 0.0357
1,000		200	41 (–18%)	
Male				
0	500	200	39	*p* = 0.0512
	1,000			*p* = 0.0001
500	1,000	200	36 (–7.69%)	*p* = 0.1132
1,000		200	31 (–20.51%)	

Cohort sizes, mean and median lifespans, percentage changes, and log-rank (Mantel-Cox) tests for survival curves in this study.

Male *D. melanogaster* exhibited distinct lifespan responses to PS exposure compared to females (Figure 3B and Table 1). Both 500 mg/L and 1,000 mg/L PS significantly reduced males’ longevity compared to controls (*p* < 0.0001 for both). However, while 500 mg/L PS exposure showed only a marginal, non-significant reduction in median lifespan (7.7% decrease; 36 vs. 39 days, *p* = 0.051, HR = 1.39, 95% CI [1.42, 1.70]), 1000 mg/L PS caused a robust 20.5% reduction (31 days, *p* = 0.0001, HR = 1.54, 95% CI [1.26, 1.89]). Interestingly, the difference between 500 and 1,000 mg/L exposures was not statistically significant (*p* = 0.113, HR = 1.21, 95% CI [0.99, 1.47]), indicating a potential threshold effect for male lifespan reduction.

### Gut microbiota

To assess PS-induced alterations in gut microbiota composition and trans-generational effects, we performed 16S rRNA gene amplicon sequencing on both control (0 mg/L PS) and 1,000 mg/L PS-exposed flies, along with their offspring. Our sequencing generated 4,201,752 high-quality reads. After DADA2 denoising and chimera removal, all samples retained ≥ 10,000 high-quality reads (median = 168,698; range = 10,8229–181,929). These results revealed considerable microbial diversity, with ASVs per sample ranging from 81 to 518. The complete 16S rRNA gene sequencing dataset (raw fastq) has been deposited in the NCBI Sequence Read Archive (SRA) under accession number PRJNA1216220, ensuring full data accessibility for future research.

### Effects of PS on the gut microbial composition of flies

**FIGURE 4:**
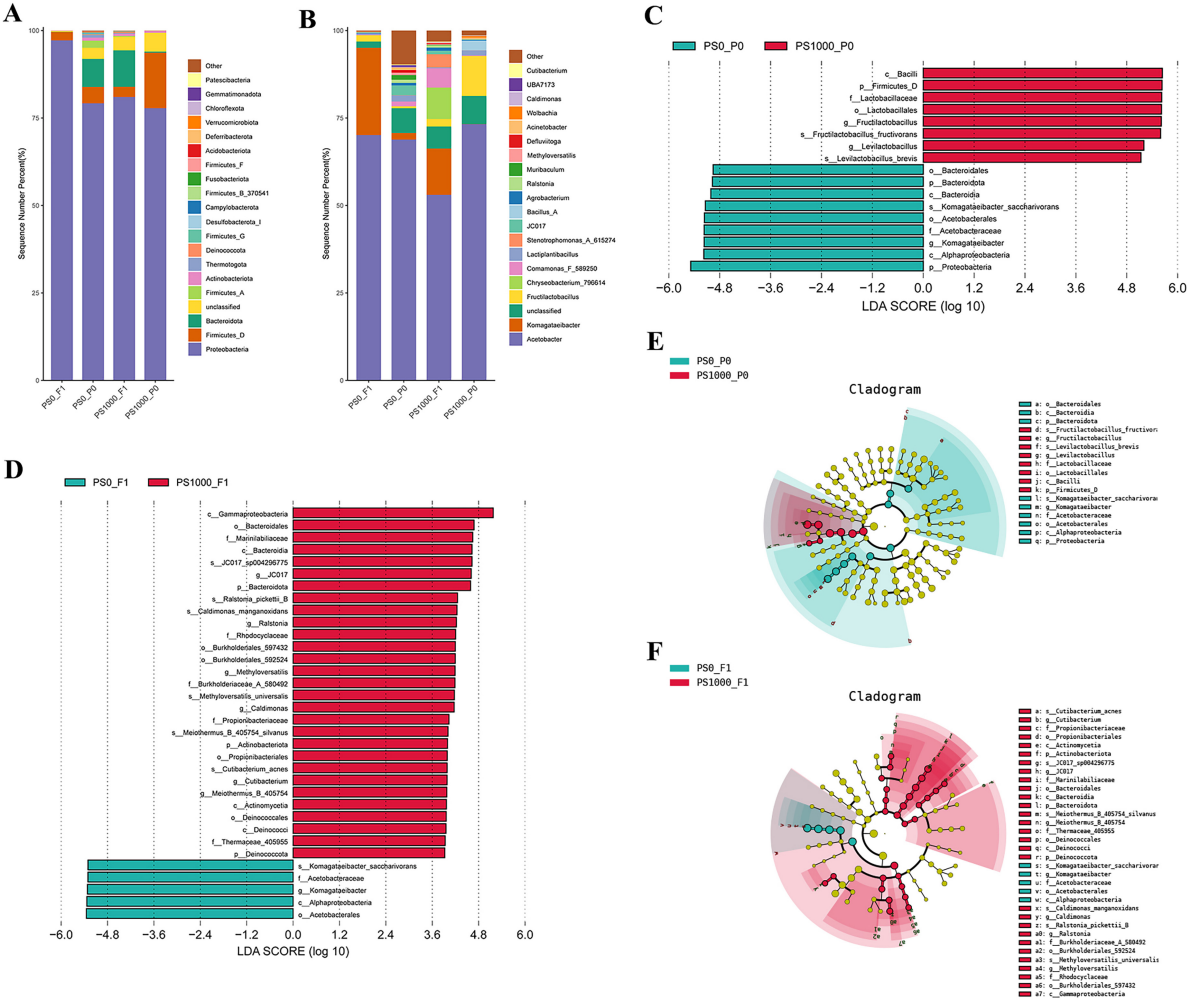
Gut microbial composition and species enrichment analysis. (A) Gut microbial composition at phylum level. (B) Gut microbial composition at genus level. (C) Linear discriminant analysis (LDA) scores of gut microbial species in 0 mg/L and 1,000 mg/L PS-exposed flies. (D) LDA scores of gut microbial species in offspring of 0 mg/L and 1,000 mg/L PS-exposed flies. (E) Cladogram of enriched species in 0 mg/L and 1,000 mg/L PS-exposed flies. (F) Cladogram of enriched species in offspring of 0 mg/L and 1,000 mg/L PS-exposed flies.

Microbiome profiling revealed consistent phylum-level composition across all experimental groups, with Proteobacteria, Firmicutes, and Bacteroidota collectively representing > 85% of relative abundance in both 1,000 mg/L PS-exposed flies and controls (0 mg/L PS), and their offspring (Figure 4A). This conservation was maintained at the genus level, where *Acetobacter*, *Komagataeibacter*, and *Fructilactobacillu*s constituted the core microbiota (> 70% relative abundance) across all groups (Figure 4B).

LEfSe analysis identified significant (LDA score > 2, *p* < 0.05) taxonomic shifts associated with PS exposure (Figures 4C,E). Control flies exhibited enrichment of Firmicutes-associated taxa, including Lactobacillaceae, *Komagataeibacter saccharivorans*, Acetobacterales, and Acetobacteraceae. In contrast, 1,000 mg/L PS-exposed flies showed predominant enrichment of Proteobacteria- and Bacteroidota-affiliated taxa, particularly *Fructilactobacillus, Levilactobacillus brevis, Levilactobacillus, and Fructilactobacillus fructivorans*.

LEfSe analysis of offspring microbiota revealed distinct, parentally transmitted enrichment patterns associated with PS exposure (LDA score > 2.0, *p* < 0.05; Figures 4D,F). Control offspring (0 mg/L PS parents) showed selective enrichment of Proteobacteria-associated taxa, including: Acetobacterales, Acetobacteraceae, *Komagataeibacter*, and *Komagataeibacter saccharivorans*. In contrast, offspring from 1,000 mg/L PS-exposed parents exhibited enrichment of phylogenetically diverse taxa spanning four phyla (Proteobacteria, Bacteroidota, Actinobacteriota, and Deinococcota): *Caldimonas manganoxidans*, *Cutibacterium acnes*, *Corynebacterium tuberculostearicum*, *Methyloversatilis*, Thermaceae 405955, Rhodocyclaceae, *Caldimonas*, *Cutibacterium*, *Meiothermus B 405754 silvanus*, *JC017 sp004296775*, *Ralstonia*, *Ralstonia pickettii* B, and *Methyloversatilis universalis*. This expansion included both environmental (e.g., *Ralstonia pickettii*) and host-associated (e.g., *Cutibacterium*) species, suggesting significant ecological restructuring of the offspring microbiome.

These results reveal a profound trans-generational effect of PS exposure, establishing two distinct microbial ecosystems in offspring: (1) control offspring maintain the characteristic *Drosophila* Proteobacteria-dominated profile; (2) while PS-lineage offspring develop a significantly diversified microbiota spanning four bacterial phyla. This dichotomy demonstrates that parental PS exposure fundamentally restructures offspring gut microbial communities across multiple taxonomic levels, potentially altering microbiome-mediated host functions.

**FIGURE 5:**
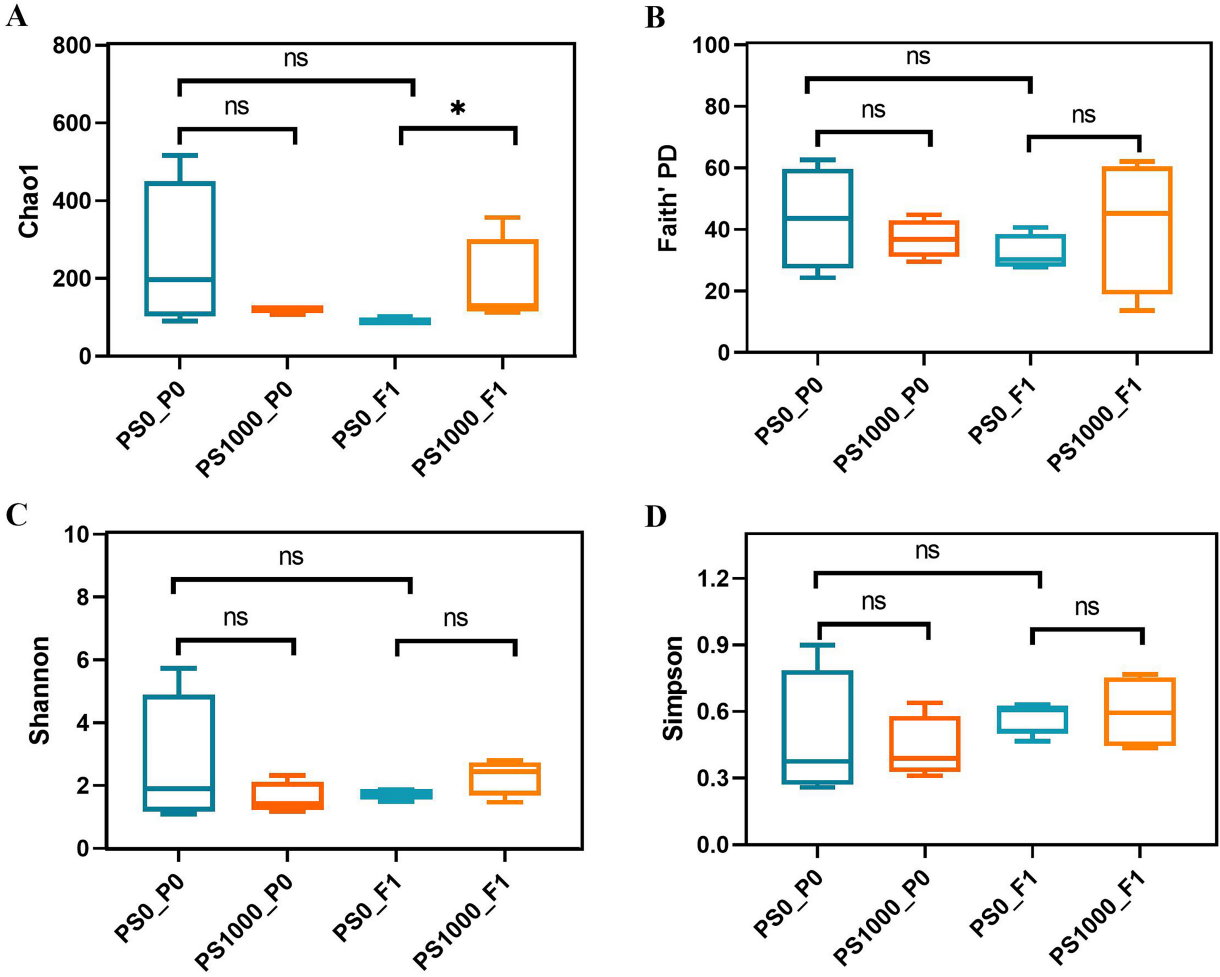
α diversity of gut microbiota in PS-exposed flies and their offspring. (A) Chao1. (B) Faith’s PD. (C) Shannon. (D) Simpson. *p < 0.05.

Alpha diversity analysis demonstrated selective trans-generational impacts of PS exposure on gut microbial communities (Figure 5). While direct exposure to 1,000 mg/L PS did not alter gut microbiota richness (representing by Chao1 index) compared to controls (0 mg/L PS), offspring of PS-exposed flies exhibited significantly increased Chao1 index, indicating enhanced microbial richness (Figure 5A). However, neither direct PS exposure nor trans-generational effects influenced other diversity metrics: Faith,s phylogenetic diversity (PD), Simpson index, and Shannon index remained comparable across all groups (Figures 5B–D). These findings reveal that parental PS exposure specifically amplifies microbial richness in offspring (Chao1), without affecting community phylogenetic breadth (Faith’s PD) or evenness (Simpson/Shannon indices), suggesting targeted rather than global modifications of microbial diversity.

**FIGURE 6:**
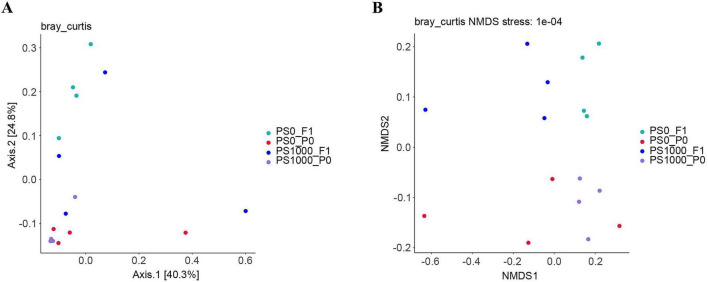
β diversity of gut microbiota in PS-exposed flies and their offspring. **(A)** Principal coordinate analysis (PCoA) score plot based on bray-curtis distance. **(B)** Non-metric multidimensional scaling (NMDS) score plot based on bray-curtis distance.

β-diversity analysis demonstrated structural conservation of gut microbial communities following PS exposure (Figures 6A,B and Table 2). Neither direct exposure to 1,000 mg/L PS in adult flies (PERMANOVA, *p* = 0.125) nor trans-generational transmission to offspring (PERMANOVA, *p* = 0.123) significantly altered overall microbiota composition. While non-metric multidimensional scaling (NMDS) revealed visual separation between parental and offspring microbiomes, this clustering pattern did not reach statistical significance (PERMANOVA, *p* = 0.123), indicating preserved community structure across generations despite PS exposure. The microbial species were presented in ASV table (Supplementary Table 2).

**TABLE 2:** Permanova of microbiota based on bray-curtis distance.

Group 1	Group 2	Sample size	Permutations	Pseudo-*F*	*p*-value	*q*-value
**PS0_P0**	PS1000_P0	8	999	1.527494322	0.125	0.15
	PS0_F1	8	999	3.777115031	0.035	0.102
**PS0_F1**	PS1000_F1	8	999	1.491530122	0.123	0.15

PS0_P0 represents PS-exposed parental flies, PS0_F1 represents offspring flies from PS-exposed parental flies.

### Effects of PS on gut microbial function of flies

**FIGURE 7:**
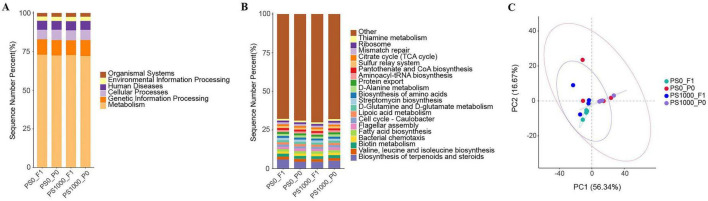
Function prediction of gut microbiota in PS-exposed flies and their offspring. **(A)** KEGG pathway at L1 level. **(B)** KEGG pathway at L3 level. **(C)** Principal Component Analysis (PCA) of gut microbial function at L3 level.

Functional profiling of gut microbiota revealed conserved metabolic potential across treatment groups (Figure 7). At KEGG Level 1 (L1), metabolic pathways constituted primary of predicted functions across all groups (Figure 7A). Level 3 (L3) analysis identified 19 core functional modules (> 1% relative abundance each), including: Amino acid metabolism (valine/leucine/isoleucine and D-glutamine/D-glutamate biosynthesis); Cofactor/vitamin metabolism (biotin, lipoic acid, thiamine, and pantothenate/CoA biosynthesis); Energy metabolism (TCA cycle, fatty acid biosynthesis); Cellular processes (bacterial chemotaxis, flagellar assembly, cell cycle regulation); and Genetic information processing (aminoacyl-tRNA biosynthesis, protein export, ribosome, mismatch repair) (Figure 7B). Principal Component Analysis (PCA) further confirmed functional stability, showing no significant alteration of functional profile of the gut microbiota across both PS-exposed flies and their offspring groups (Figure 7C).

**FIGURE 8:**
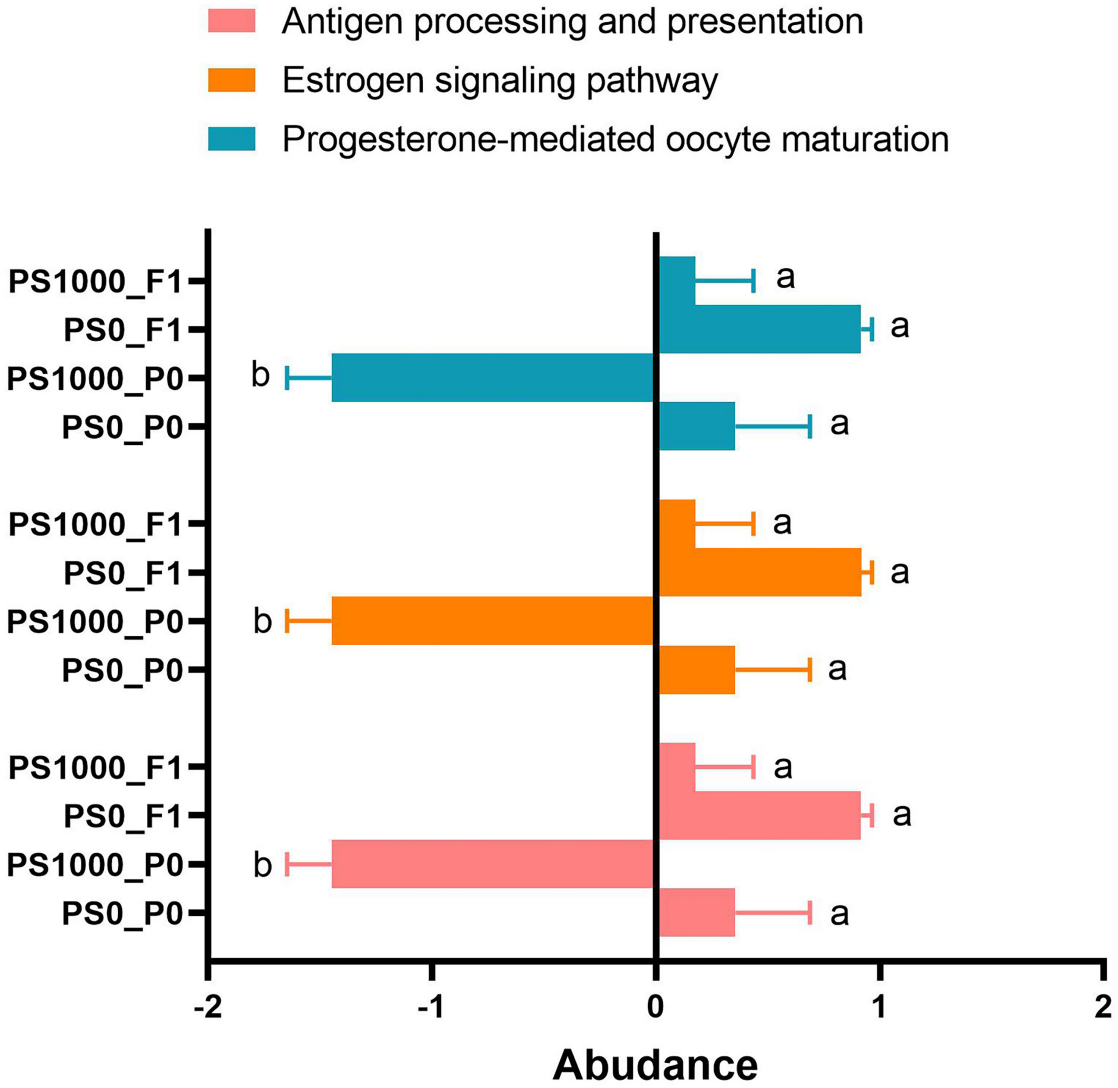
Significant pathway (ANOVA analysis) of gut microbial function in PS-exposed flies and their offspring. Here, the same letters (a, b) above the bars indicate that there is no significant difference between the groups sharing the same letter. Conversely, different letters above the bars denote a significant difference between the corresponding groups.

Targeted analysis of host-microbe interaction pathways revealed significant PS-induced alterations in parental generations (Figure 8). Compared to controls (0 mg/L PS), adult flies exposed to 1,000 mg/L PS showed marked reductions in three critical functional pathways: Antigen processing and presentation, Estrogen signaling, and Progesterone-mediated oocyte maturation. Notably, these pathway alterations were not observed in the offspring generation, demonstrating trans-generational functional recovery in offspring microbiota. These results suggest that while high-dose PS exposure acutely impairs host-interactive microbial functions, these effects are not inherited by subsequent generations.

### Related gene expressions of PS-exposed flies

**FIGURE 9:**
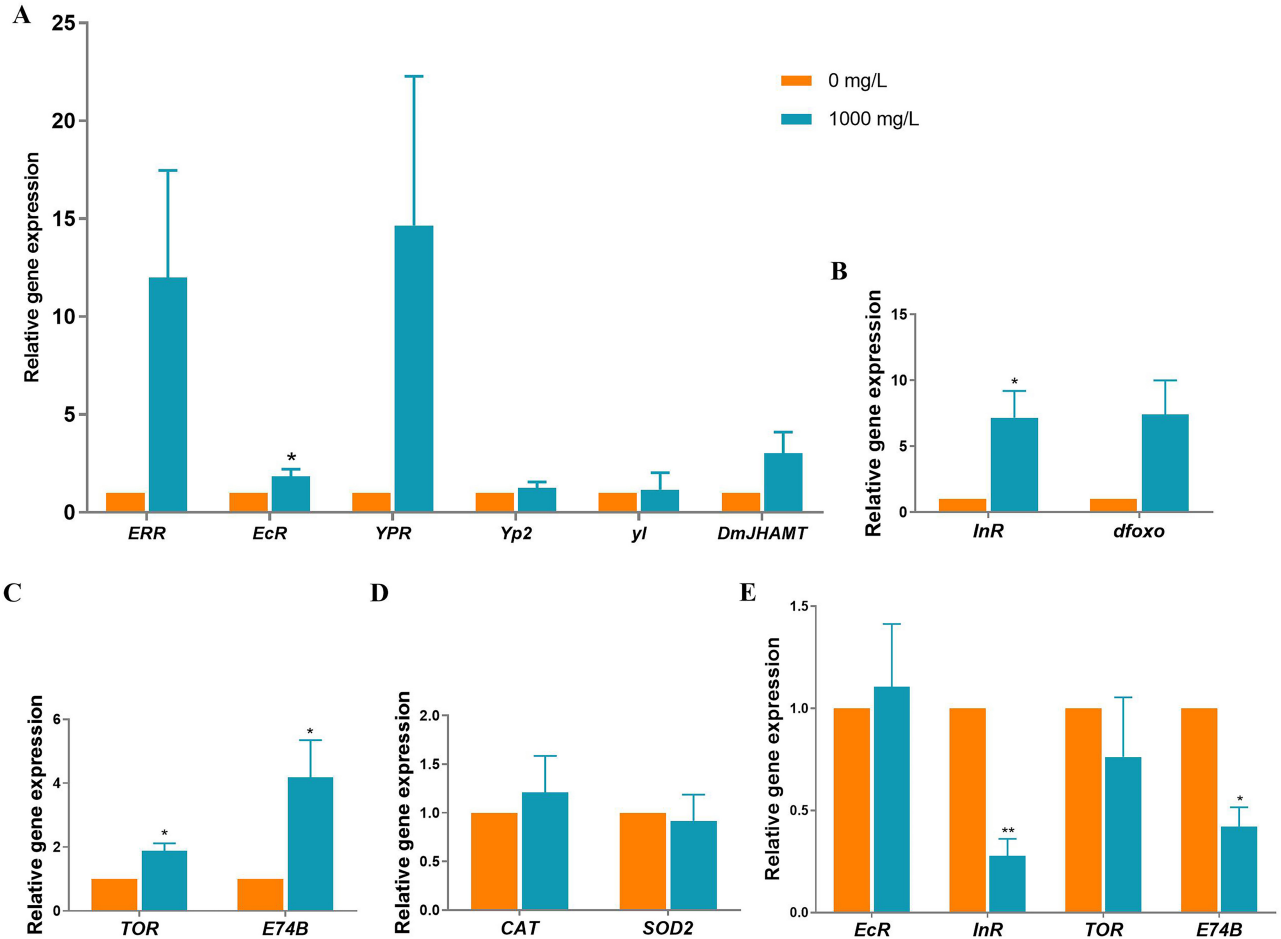
Gene expressions of flies exposed to PS. **(A)** Hormone-related genes. **(B)** IIS pathway-related genes. **(C)** TOR pathway-related genes. **(D)** Antioxidant enzymes-related genes. **(E)** Changed gene expressions of offspring from PS-exposed flies. *p < 0.05, **p < 0.01.

Gene expression analysis revealed distinct trans-generational impacts of PS exposure on endocrine and metabolic pathways (Figure 9). In directly exposed adult parent flies, the hormone-related gene of *EcR* mRNA level significantly increased in 1,000 mg/L PS-exposed flies compared to 0 mg/L PS-exposed flies (Figure 9A). However, the mRNA transcripts of other genes, including *ERR*, *YPR*, *Yp2*, *yl* and *DmJHAMT*, were not significantly different between 0 and 1,000 mg/L PS-exposed flies (Figure 9A). For IIS pathway-related genes *InR* and *dfoxo*, the mRNA transcript of *InR* significantly increased in 1,000 mg/L PS-exposed flies, whereas, the mRNA transcript of *dfoxo* was unchanged (Figure 9B). For TOR pathway-related genes, mRNA transcripts of both *TOR* and *E74B* were significantly increased in 1,000 mg/L PS-exposed flies (Figure 9C). For antioxidant enzymes-related genes, neither *CAT* nor *SOD2* was changed by 1,000 mg/L PS (Figure 9D).

Trans-generational analysis revealed selective persistence of metabolic disruptions in offspring (Figure 9E). While the PS-induced upregulation of *EcR* and *TOR* observed in parents was not maintained in offspring, suggesting differential stability of metabolic programming across generations. However, two key metabolic regulators showed heritable suppression: *InR* and *E74B*, which were significantly decreased in offsprings. The pattern suggested that pathway-specific trans-generational inheritance, and potential metabolic trade-offs in offspring despite recovery of some parental perturbations.

## Discussion

In modern society, humans and ecosystems are increasingly exposed to synthetic chemicals, including food additives like potassium sorbate (PS, E202). Widely used in food, pharmaceuticals, cosmetics, and animal feed, PS is valued for its strong preservative efficacy, low toxicity, and minimal impact on product characteristics (Dehghan et al., 2018; WHO/FAO Joint Expert Committee, 2016). It is efficiently metabolized to CO_2_ and water with negligible accumulation in the body, supporting its general recognition as safe (EFSA Panel on Food Additives and Nutrient Sources added to Food [ANS], 2015). However, rising consumption of processed foods has intensified concerns about chronic and cumulative exposure, particularly during sensitive developmental windows such as early childhood. Despite its widespread use, research on PS’s effects on development, gut microbiota establishment, and trans-generational impacts remains limited.

Previous studies on PS have reported inconsistent findings, often constrained by the use of animal models without focusing on the early-life stage or *in vitro* systems. For instance, 10 weeks PS exposure (150–1,000 mg/kg feed) induced hepatic inflammation and elevated IL-1β in 5–6 weeks-old mice (Xiao et al., 2024), while lower doses (25 mg/kg bw/day) activated inflammatory pathways via *NF*κ*B* and *MAPK8* in 6–8 weeks-old mice (Raposa et al., 2016). Conversely, 6–8 weeks old zebrafish exposed to PS (0.1–1.0 g/L) showed reduced immune markers, including IL-1β and TNF-α (Peng et al., 2019), highlighting model- and dose-dependent variability. Genotoxicity studies are similarly conflicting: PS increased chromosomal aberrations in human lymphocytes at high concentrations (Mamur et al., 2010), but caused no DNA fragmentation in endothelial cells at lower doses (Mohammadzadeh-Aghdash et al., 2018). These discrepancies underscore the need for early life stage developmental-stage-specific assessments.

In this study, we employed *D. melanogaster* to investigate PS effects across the lifespan, focusing on larval development, adult longevity, and trans-generational outcomes. We observed a biphasic, dose-dependent effect: 25 mg/L PS accelerated larval development and adult emergence, while ≥ 500 mg/L delayed these processes. This is an interesting finding different from previous studies, in which no prior studies reported a promotional effect of PS was reported. These promotional effects require confirmation. It is also necessary to confirm whether the observed promoting effect in 25 mg/L PS group is the synergistic effect of PS and methylparaben in food of *D. melanogaster*.

Interestingly, the retarded pupation time was found in F1 flies, indicating a trans-generational effect of PS on *D. melanogaster* development and merit further investigation. Our results also showed that the female’s lifespan was significantly decreased in 1,000 mg/L PS-exposed flies. Male lifespan was reduced in both 500 mg/L and 1,000 mg/L PS-exposed flies. The results of PS effect on lifespan was consistent to a recent study by Zhang et al. (2024) assessing the PS effect on lifespan and fecundity also using a *Drosophila* model, in which PS at concentrations of 0.05, 0.1, 0.5, and 5% reduced the lifespan and fecundity of *Drosophila*. However, Zhang et al. (2024) also did not investigate any of the larval development.

Gut microbiota analysis in our study revealed that 1,000 mg/L PS altered taxonomic composition despite no major shifts in α- or β-diversity. These results were some different from results reported in previous studies. Peng et al. (2019) discovered that exposing zebrafish to 0.1 and 1.0 g/L aqueous solutions of PS led to some destabilization of the microbiome composition. This included a reduction in the abundance of specific bacterial genera as well as alterations in the relative levels of transcription and carbohydrate metabolism associated with microbial reproductive ability and activity. Hrncirova et al. (2019a) investigated the susceptibility of gut microbiota to sodium benzoate, sodium nitrite, and PS using a broth microdilution method. Their findings indicated that gut bacteria with anti-inflammatory properties, such as *Clostridium tyrobutyricum* or *Lactobacillus paracasei*, were significantly reduced by PS compared to intestinal bacteria with pro-inflammatory or colitogenic properties, such as *Bacteroides taiotaomicron* or *Enterococcus faecalis* (Hrncirova et al., 2019a). Hrncirova et al. (2019b) further found that a mixture of commonly used antimicrobial food additives at exposure levels of 4.8 mg/kg bw/day for benzoate, 0.36 mg/kg bw/day for nitrite, and 19.0 mg/kg bw/day for sorbate induced dysbiosis in the human gut microbiota. This dysbiosis was characterized by a decrease in microbiota diversity, a depletion of the Clostridiales order, and an expansion of the Proteobacteria phylum. However, this study did not investigate the functional changes in the altered gut microbiota. A 12-week experiment observed increased proportions of *Parabacteroides* and *Adlercreutzia* by PS at concentrations of 500 mg/L (0.007 mg/kg bw/day) (Nagpal et al., 2021). A recent study also observed changes in gut microbiota abundances in mice due to the intake of PS (Xiao et al., 2024). These literatures suggest that PS not only changes the gut microbiota genus but also altered the α- and β-diversity.

In our study, 1,000 mg/L PS altered genera abundance and caused occurrence of different enrichment species. Firmicutes-associated taxa abundance of Lactobacillaceae, *Komagataeibacter saccharivorans*, Acetobacterales, and Acetobacteraceae were enriched in PS-free flies; Abundance of Proteobacteria- and Bacteroidota-affiliated taxa, *Fructilactobacillus, Levilactobacillus brevis, Levilactobacillus, and Fructilactobacillus fructivorans*, exhibited enrichment in 1,000 mg/L PS-exposed flies. Changes in the abundance of gut microbiota led to alterations in its function. Antigen processing and presentation, estrogen signaling pathway, and progesterone-mediated oocyte maturation were significantly decreased in 1,000 mg/L PS-exposed flies compared to 0 mg/L PS-exposed flies. Estrogen and progesterone were important endocrine hormones in *D. melanogaster* growth and development (Tennessen and Thummel, 2011; Tennessen et al., 2011). The decreased estrogen signaling pathway and progesterone-mediated oocyte maturation suggesting that the delayed larval development by PS may associate with gut microbiota and it altered pathways in *D. melanogaster*. These results suggested PS may alter the establishment and development of gut microbiota in *D. melanogaster* larvae during intervention, leading to the formation of a relatively distinct changed enrichment genera species in adult flies, these altered gut microbiota then further changed the development of *D. melanogaster*.

However, PS did not dramatically change α diversity indexes and β diversity indices, which may because the limited sample size, sequencing depth, compositional shifts restricted to low-abundance ASVs, or taxonomy-level changes that don’t alter diversity indices. Another explanation was the ways of PS absorption and metabolism pathways in organism. The results of metabolic studies indicate that PS is fully absorbed following oral administration and subsequently distributed throughout the body. Specifically, 80–86% of the substance is exhaled as CO_2_ through the lungs, while 2–10% is excreted via urine primarily as urea, and in lower concentrations as muconic and sorbic acids. The lung excretion process is typically completed within 10 h of ingestion (Taghavi et al., 2013). In another study, it is reported that 85% of sorbic acid was metabolized into CO_2_ through the lungs, with 3% remaining in internal organs and another 3% found in skeletal muscles. Approximately 2% was excreted in the urine as urea, while only 0.4% was excreted in the feces. The remaining 6.6% was distributed in other parts of the body (Dehghan et al., 2018). Both studies indicate that the amount of sorbic acid remaining in the intestine is extremely low. Thus, we speculate that the very small amount of PS remaining in the intestine does not directly significantly affect the composition of the gut microbiota.

Notably, the offspring of PS-exposed parents exhibited more pronounced gut dysbiosis than their parents, with higher Chao1 richness and distinct microbial enrichment, despite no direct exposure. We speculated that PS may not directly cause changes in the gut microbiota but rather indirectly through other mechanisms. Such trans-generational microbiome effects have not been previously documented for PS and merit further investigation.

The trans-generational developmental delay in offspring of PS–exposed flies may result from the following ways: stress-induced epigenetic changes in parental gametes, or vertical transmission of an altered microbiome based on the sorbate’s antimicrobial properties, or reduced or modified maternal provisioning due to metabolic disruption. Given the property of water-soluble, PS is unlikely to persist in tissues, suggesting effects are mediated by physiological or molecular memory rather than direct germline contamination. Future work should use cross-fostering to separate egg-intrinsic from post-ovipositional effects, microbiota transplantation into germ-free larvae to test microbial causality, and epigenetic profiling of gametes to identify heritable regulatory changes.

Molecular mechanistically, we found upregulated expression of key developmental genes in 1,000 mg/L PS-exposed flies: *EcR*, *InR*, *TOR*, and *E74B*. The Ecdysone Receptor (EcR) is a nuclear hormone receptor that activates arthropod steroid hormones known as ecdysteroids. It plays a crucial role in regulating key biological processes in insects, including molting, metamorphosis, reproduction, diapause, and innate immunity (Koelle et al., 1991; Yao et al., 1992; Thomas et al., 1993). EcR is particularly important for the larval-to-prepupal transition in *Drosophila* (Uyehara and McKay, 2019). Different isoforms of EcR are essential for larval molting and neuron remodeling during insect metamorphosis (Schubiger et al., 1998; Xu et al., 2020). Moreover, numerous studies have highlighted EcR’s pivotal role in affecting lifespan and reproduction (Antoniewski et al., 1996; Thummel, 1996; Riddiford et al., 2000). In our study, the mRNA transcripts of *EcR* significantly increased in 1,000 mg/L PS-exposed flies. Given previous research indicating that reduced *EcR* can prolong adult longevity and stress resistance in flies (Simon et al., 2003; Tricoire et al., 2009), the elevated *EcR* levels in our study may be one of the reasons for the developmental delay observed in 1,000 mg/L PS-exposed flies. The unchanged expression of *EcR* in the offspring of 1,000 mg/L PS-exposed flies suggests that *EcR* levels returned to normal in these offspring. Other hormone-related genes were also tested in our study. For example, the estrogen-related receptor (ERR) coding gene *ERR*, the yolk protein (YP) receptor (YPR) coding gene *YPR*, juvenile hormone (JH) acid O-methyltransferase (JHAMT) coding gene *DmJHAMT*, and yolkless coding gene *yl* were unchanged in 1,000 mg/L PS-exposed flies. This suggests that the delay in growth and development of 1,000 mg/L PS-exposed flies may not be regulated by these genes.

The Insulin/IGF-1 Signaling (IIS) pathway is another critical factor that can affect *D. melanogaster* development. Research has shown that the IIS pathway controls larval formation, stress resistance, and longevity (Kapahi et al., 2004). Specifically, the IIS pathway directly regulates the timing of *D. melanogaster* developmental transitions by influencing the production of the molting hormone ecdysone (Ghosh, 2021). Previous study had indicated that increased IIS activity accelerates larval growth rates and promotes *D. melanogaster* metamorphosis (Walkiewicz and Stern, 2009). In our study, exposure to 1,000 mg/L PS led to an unexpected increase in insulin receptor (*InR*) mRNA levels in adult flies, despite their significantly delayed developmental time. Given that elevated *InR* expression typically promotes growth and accelerates development in *Drosophila* (Shingleton et al., 2005), this observation suggests a potential decoupling between *InR* transcript abundance and functional insulin signaling, which possibly due to post-transcriptional regulation, receptor desensitization, or impaired downstream pathway activation under PS stress. In contrast, the offspring of PS-exposed flies exhibited both reduced *InR* expression and delayed development, consistent with the canonical role of insulin signaling in regulating developmental timing. This transgenerational suppression of *InR* may therefore contribute directly to the observed developmental delay in the F1 offspring.

The Target of Rapamycin (TOR) pathway has emerged as a key regulator of growth and size in *D. melanogaster* (Kapahi et al., 2004). This highly conserved nutrient-sensing pathway plays crucial roles in regulating growth, metabolism, and aging (Bjedov et al., 2010). It interacted with *Lactobacillus plantarum* influences both *InR* and Ecdysone signaling in larvae. In this study, we found increased expressions of the mRNA transcripts of *TOR* and *E74B* in 1,000 mg/L PS-exposed flies, suggesting that the delayed developmental time may result from the upregulation of *TOR* and *E74B*. In the offspring of 1,000 mg/L PS-exposed flies, the mRNA transcripts of *TOR* returned to normal levels; however, the decreased expression of *E74B* still requires further investigation.

While this study provides novel insights into the potential developmental and trans-generational toxicity of PS, several limitations should be considered. First, although *D. melanogaster* is a useful model for toxicity assessment, its relevance to human development is constrained by fundamental differences in anatomy, endocrine regulation, and immune function between insects and mammals. Second, we delivered PS through fly food, whereas a primary route of human exposure, especially in early childhood, is through contaminated water and beverages; the bioavailability and local gastrointestinal interactions of PS may differ substantially between these matrices. Third, the highest concentration (2,000 mg/L) used in this study far exceed typical human dietary exposure, suggesting our findings under the context of high PS concentration reflect biological hazard and possible mechanisms rather than realistic risk. Finally, although we observed persistent developmental delays and microbiome shifts across generations, the underlying causes remain unclear. Future work should test whether transferring the altered microbiota to germ-free larvae reproduces the phenotype and investigate whether PS exposure disrupts endocrine signaling through direct or indirect pathways. And clarify the role of the gut microbiota using metagenomic and metabolomic sequencing approaches.

In summary, our study reveals that PS exerts dual-phase developmental toxicity linked to endocrine disruption, with attenuated but persistent effects across generations. Low-dose exposure (25 mg/L) accelerates developmental timing, while higher doses (≥ 500 mg/L) cause significant developmental delays and reduced lifespan, associated with dysregulation of key endocrine and nutrient-sensing pathways (*EcR*, *InR*, *TOR*, and *E74B*). Notably, developmental delays persist in the unexposed F1 offspring, indicating trans-generational toxicity. Intriguingly, while gut microbiota remains largely stable in exposed parents, their offspring exhibit pronounced dysbiosis, despite near-complete recovery of gene expression, suggesting that trans-generational microbiome alterations may occur through mechanisms independent of direct transcriptional disruption in the host. These findings highlight the potential for PS to induce lasting biological effects across generations, even after cessation of exposure. However, mechanistic experiments are needed to disentangle direct host endocrine effects from microbiome–mediated mechanisms and to assess the relevance of these findings to vertebrate models and human exposure scenarios.

## Data Availability

The original contributions presented in the study are publicly available. This data can be found at the National Center for Biotechnology Information (NCBI) using accession number PRJNA1216220, https://www.ncbi.nlm.nih.gov/bioproject/PRJNA1216220/.
