# Supplementary material for: Potassium sorbate induces developmental and microbiome changes in Drosophila melanogaster with attenuated trans-generational toxicity
Source: Front Microbiol. 2026 Mar 23;17:1783630. doi: 10.3389/fmicb.2026.1783630 (PMC13050859; doi:10.3389/fmicb.2026.1783630)
Supplement: Supplementary file 1 [file Data_Sheet_1.pdf]

Table S1. Primer Sequences of qRT-PCR used in this study.

Gene	Primer sequences (5'→3')	Reference
ERR	CCTGAAATCCTCGCCCTC;<br>CCATTGCCGCCACTTGTA	(Liu, Li, Zhao, Zhang, & Gu, 2014)
EcR	AAGGAAGGTATATTGCGCGC;<br>GGAGAACCAATGTGCGATGA	(Liu et al., 2014)
YPR	AGCAGCCATGCGCTCAGCTA;<br>GCATTACGGGTTGTTCGCTCAGG	(Liu et al., 2014)
Yp2	GGCGCTACGCTGTTGGA;<br>GGGCTTGAAGACATGGTTGAA	(Bovier, Rossi, Mita, & Digilio, 2018)
yl	CGAACGAGGATGCTTAAATCTATGT;<br>CGGTTCCGGCATAGGAATT	(Bovier et al., 2018)
DmJHAMT	AAACATATGAATCAGGCCTCTCTATATCAG;<br>AACTCGAGGACTCTGTTAACAAATGCAATTACTG	(Niwa et al., 2008)
InR	AACAGTGGCGGATTCGGTT;<br>TACTCGGAGCATTGGAGGCAT	(Obata, Fons, & Gould, 2018)
dfoxo	TCGAGTGCAATGTCGAGGAG;<br>AGCGGTATATTGATGTCCAGCAG	(Boyd et al., 2011)
Tor	GCTCAGAGGCGAGAGACAAG;<br>CCAGCTCACGGAGGATAAAG	(Boyd et al., 2011)
E74B	GAATCCGTAGCCTCCGACTGT;<br>AGGAGGGAGAGTGGTGGTGTT	(Obata et al., 2018)
cat	CCTCTGATTCCTGTGGGCAA;<br>GACGACCATGCAGCATCTTG	(Staats et al., 2018)
sod2	AATTTCGCAAACTGCAAGC;<br>TGATGCAGCTCCATGATCTC	(Obata et al., 2018)
Rp49	CAGTCGGATCGATATGCTAAGC;<br>GGCATCAGATACTGTCCCTTGAA	(Bovier et al., 2018)

References

Bovier, T. F., Rossi, S., Mita, D. G., & Digilio, F. A. (2018). Effects of the synthetic estrogen 17-α-ethinylestradiol on *Drosophila melanogaster*: Dose and gender dependence. *Ecotoxicology and environmental safety, 162*, 625-632.

Boyd, O., Weng, P., Sun, X., Alberico, T., Laslo, M., Obenland, D. M., . . . Zou, S. (2011). Nectarine promotes longevity in *Drosophila melanogaster*. *Free Radical Biology and Medicine, 50*(11), 1669-1678.

Liu, T., Li, Y., Zhao, X., Zhang, M., & Gu, W. (2014). Ethylparaben affects lifespan, fecundity, and the expression levels of *ERR*, *EcR* and *YPR* in *Drosophila melanogaster*. *Journal of insect physiology, 71*, 1-7.

Niwa, R., Niimi, T., Honda, N., Yoshiyama, M., Itoyama, K., Kataoka, H., & Shinoda, T. (2008). Juvenile hormone acid O-methyltransferase in *Drosophila melanogaster*. *Insect biochemistry and molecular biology, 38*(7), 714-720.

Obata, F., Fons, C. O., & Gould, A. P. (2018). Early-life exposure to low-dose oxidants can increase longevity via microbiome remodelling in *Drosophila*. *Nature Communications, 9*(1), 975.

doi:10.1038/s41467-018-03070-w

Staats, S., Wagner, A., Kowalewski, B., Rieck, F., Soukup, S., Kulling, S., & Rimbach, G. (2018). Dietary resveratrol does not affect life span, body composition, stress response, and longevity-related gene expression in *Drosophila melanogaster*. *International Journal of Molecular Sciences, 19*(1), 223.
